# Programmed death-ligand-1 expression in advanced gastric cancer detected with RNA *in situ* hybridization and its clinical significance

**DOI:** 10.18632/oncotarget.9381

**Published:** 2016-05-15

**Authors:** Jiajia Yuan, Jie Zhang, Yan Zhu, Na Li, Tiantian Tian, Yang Li, Yanyan Li, Zhongwu Li, Yumei Lai, Jing Gao, Lin Shen

**Affiliations:** ^1^ Department of Gastrointestinal Oncology, Key Laboratory of Carcinogenesis and Translational Research (Ministry of Education/Beijing), Peking University Cancer Hospital and Institute, Beijing, China; ^2^ Department of Thoracic Oncology II, Key Laboratory of Carcinogenesis and Translational Research (Ministry of Education/Beijing), Peking University Cancer Hospital and Institute, Beijing, China; ^3^ Advanced Cell Diagnostics, Inc., Beijing, China; ^4^ Department of Pathology, Key Laboratory of Carcinogenesis and Translational Research (Ministry of Education/Beijing), Peking University Cancer Hospital and Institute, Beijing, China

**Keywords:** programmed death-ligand-1, RNA in situ hybridization, immunohistochemistry, advanced gastric cancer

## Abstract

PD-L1 expression may be a predictive marker for anti-PD-1 therapeutic efficacy. No standard detection method of PD-L1 expression was available for advanced gastric cancer (AGC), which would be investigated in this study using RNA *in situ* hybridization and immunohistochemistry. Patients (*N* = 165) with AGC treated at Peking University Cancer Hospital from October 2008 to February 2013 were retrospectively studied. Tissue samples prior to chemotherapy were assessed for PD-L1 expression using RNA *in situ* hybridization (an RNAscope assay) and immunohistochemistry (IHC). The correlations of PD-L1 expression to patient characteristics and clinical outcomes were statistically analyzed. PD-L1 mRNA signals were located in tumor compartments or the mesenchyme in a brown dotted or clustered pattern, and PD-L1 mRNA expression in gastric cancer was heterogeneous. PD-L1-positive expressions were observed in 33.9% (56/165) and 35.1% (46/131) patients in mRNA level and protein level, respectively. A positive relationship was found between PD-L1 mRNA and PD-L1 protein, and compared to IHC, RNAscope assay could provide an intuitional and quantitative data with potential clinical application. No statistically significant differences occurred between PD-L1 expression and clinical response to chemotherapy, or survival. However, we found that PD-L1 expression was higher in intestinal type than in diffuse type. These findings suggested that the RNAscope assay may be a promising method for patient assessment in gastric cancer clinical trials, which would be illustrated in further study.

## INTRODUCTION

Gastric cancer is one of the most lethal cancers, partially due to few effective therapies. Despite substantial efforts to develop better treatments, gastric cancer prognoses, especially that which is advanced is poor [1]. To alleviate this unacceptable disease trajectory, recent studies have focused on immunotherapy of multiple cancers targetingprogrammed cell death-1 (PD-1) or programmed death-ligand-1 (PD-L1) [2, 3].

Activation of the PD-1/PD-L1 signaling pathway may cause an immunosuppressive tumor microenvironment that protects tumor cells from immune surveillance and killing [4, 5]. Thus, blocking the PD-1/PD-L1 signal pathway may be a strategy for enhancing endogenous antitumor immune effects and this can be explored in clinical trials to study the efficacy of anti-PD-1/PD-L1 therapy. Evidence suggests that anti-PD-1 therapy may improve the response rate and prognosis for patients with squamous-cell non-small cell lung carcinomas and metastatic melanoma [6–8]. Also, the efficacy of anti-PD-1/PD-L1 therapy in other cancers including gastric cancer is under study (http://www.clinicaltrials.gov/).

As a main target of anti-PD-1/PD-L1 therapy, PD-L1 expression by tumor cells may be a controversial marker for therapeutic efficacy [6, 9]. Almost all studies measured PD-L1 expression with immunohistochemistry (IHC) in formalin-fixed, paraffin-embedded (FFPE) sections, and no uniform standard was defined for PD-L1 staining. Moreover, the specificity and reproducibility of most commercially available anti-PD-L1 antibodies were uncertain and ranges of PD-L1-positive expression in the same tumor prompted different conclusions [10, 11]. Thus, an alternative method for accurately evaluating PD-L1 expression is needed.

Recently, a novel antibody-independent assay for RNA *in situ* hybridization in tumor FFPE tissues using an RNAscope assay is favored for its specificity and interpretative objectivity [12, 13]. In breast cancer and NSCLC, PD-L1 mRNA had a positive non-linear relationship with PD-L1 protein, suggesting the potential application of the RNAscope assay in future clinical studies. To provide an alternative method for PD-L1 evaluation in clinical trials of gastric cancer, PD-L1 expression in advanced gastric cancer was measured by RNAscope assay and IHC and we assessed the clinical significance.

## RESULTS

### Patient characteristics

**Figure 1:**
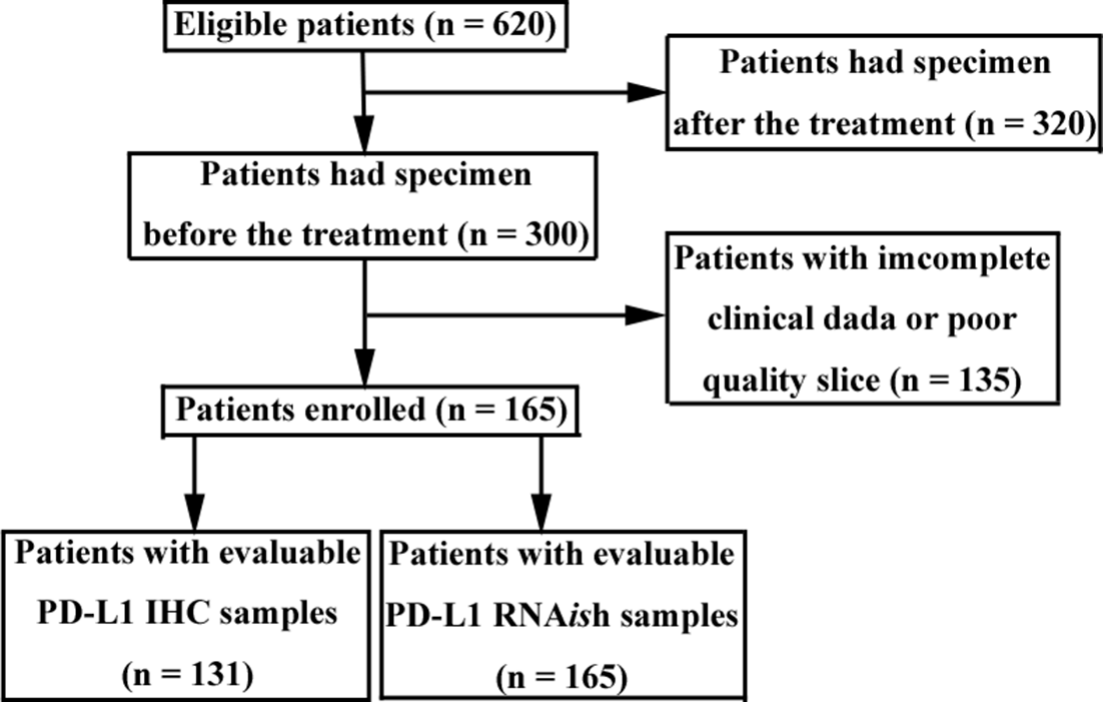
Flow chart of patient screening Eligible patients had advanced gastric cancer with tumor samples. Tumor samples were obtained by endoscopic biopsy.

Overall, 165 patients were eligible for the study and had samples evaluable for PD-L1 RNA *in situ* hybridization. Of these, 131 patients had samples evaluable for PD-L1 IHC. The screening diagram of eligible patients is depicted in Figure 1. The characteristics of all patients are shown in Table 1. The median follow-up was 63.1 months and 146 patients died (88.5%). Median overall survival (OS) was 11.8 months (95% CI = 10.2– 13.4) and median progression free survival (PFS) was 5.0 months (95% CI = 4.1–5.9).

**Table 1:** Patient characteristics

Characteristics	No. of patients (%)
Gender	
Male	134 (81.2%)
Female	31 (18.8%)
Age (years)	
≥ 65	41 (24.8%)
< 65	124 (75.2%)
KPS	
60–70	10 (6.1%)
80–100	155 (93.9%)
Differentiation^*^	
Well	37 (22.4%)
Poor	128 (77.6%)
Lauren classification	
Intestinal	23 (13.9%)
Diffuse	51 (30.9%)
Mixed	12 (7.3%)
Unknown	79 (47.9%)
Primary sites	
Non-gastroesophageal junction	98 (59.4%)
Gastroesophageal junction	67 (40.6%)
Number of metastatic organs	
≥ 3	56 (33.9%)
< 3	109 (66.1%)
Liver metastasis	
Yes	79 (47.9%)
No	86 (52.1%)
Peritoneal metastasis	
Yes	78 (47.3%)
No	87 (52.7%)

Note:

* well, including high-differentiation and middle-differentiation adenocarcinoma; poor, including low-differentiation adenocarcinoma, mucinous adenocarcinoma, and signet-ring cell carcinoma.

### PD-L1 mRNA expression and positivity threshold

**Figure 2:**
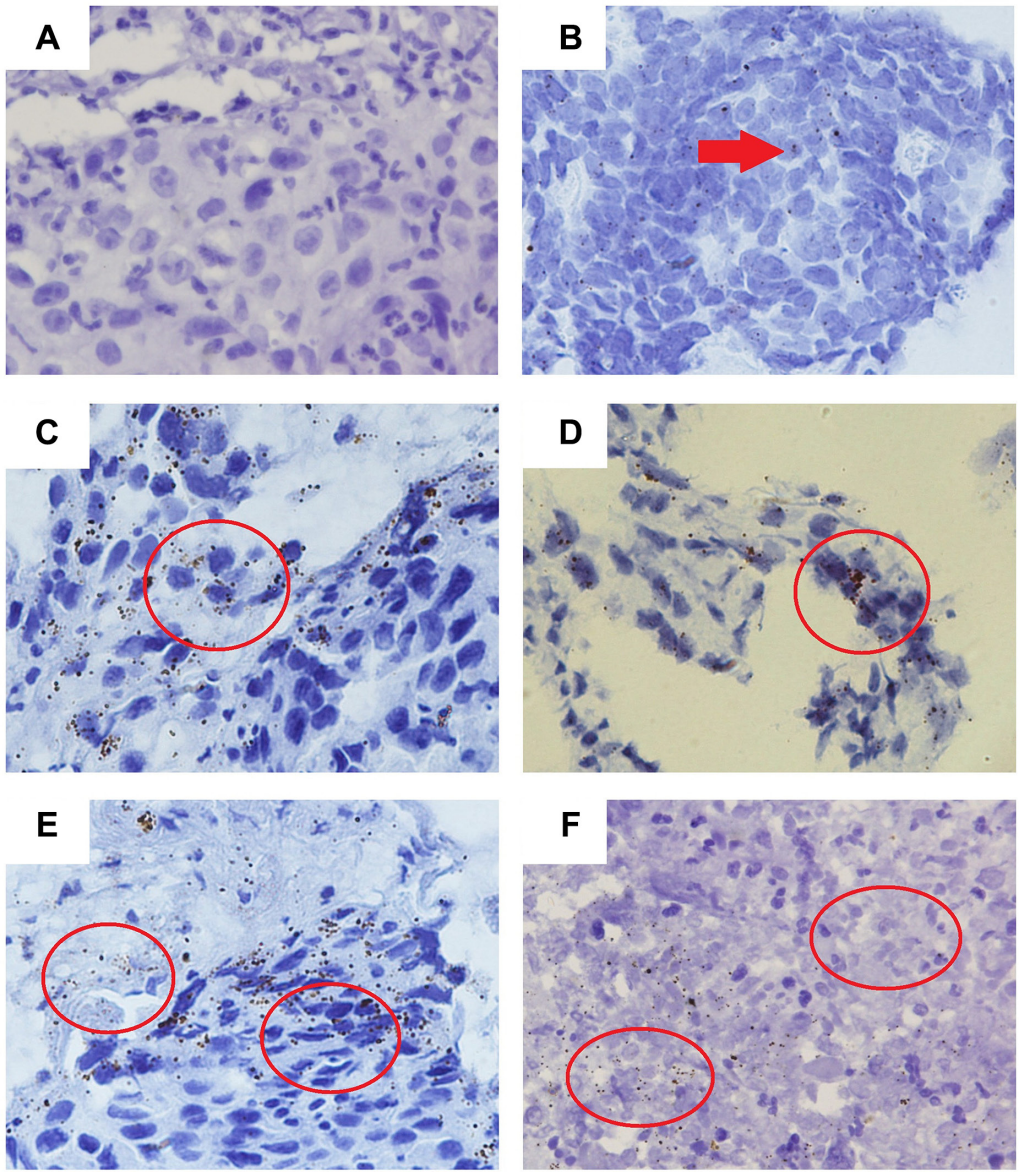
Distribution and heterogeneity of PD-L1 mRNA signals (**A**) The expression of PD-L1 mRNA signal was negative; (**B**) PD-L1 mRNA signal was located predominantly in tumor cell compartment with 0–1 dots/cell indicated by red arrow; (**C**) PD-L1 mRNA signal was identified as multiple small dots with 4–9 dots/cell as indicated by red circle; (**D**) PD-L1 mRNA signal was located in tumor compartment in clustered pattern as indicated by red circle; (**E**) PD-L1 mRNA signals were located either in tumor compartment (right red circle) or in mesenchyme (left red circle); (**F**) The heterogeneity of PD-L1 mRNA signals in one section. The left red circle indicated that PD-L1 mRNA signal was identified as multiple small dots, but the right red circle indicated that no PD-L1 mRNA signal was found.

PD-L1 mRNA signals were located in tumor compartment or mesenchyme by brown dotted or clustered patterns, and PD-L1 mRNA expression in gastric cancer was heterogeneous (Figure 2).

No standard scoring criteria for PD-L1 expression in gastric cancer was determined, so we adopted criteria from the literature. First, PD-L1 mRNA expression occurred in 33.9% patients based on positive signals. Second, the PD-L1 mRNA positivity threshold was defined as PD-L1 expression in the mesenchyme or ≥ 1% tumor cells according to criteria from the KEYNOTE-012 trial [14]. So 33.9% patients had positive expression. Third, PD-L1 mRNA-positive expression was defined as PD-L1 positive signals in ≥ 20 tumor cells based on HER2 amplification in gastric cancer [15], so 33.3% of patients were positive for this. The criteria from the KEYNOTE-012 trial was the only reported criteria of gastric cancer in the international conference on authority, we used this criteria for the following analysis in this study.

### PD-L1 protein expression and association with PD-L1 mRNA expression

Among all eligible patients, 131 patients had samples evaluable for PD-L1 IHC, and 46 (35.1%) patients presented PD-L1-positive expression. A positive relationship was found between PD-L1 mRNA and PD-L1 protein (*P* = 0.122, McNemar's test; Supplementary Figure S2), and compared to IHC, RNAscope assay could provide an intuitional and quantitative data with potential clinical application.

### Association of PD-L1 mRNA expression with clinicopathological characteristics

PD-L1 mRNA-positive and -negative expression occurred in 33.9% and 66.1% patients, respectively. No significant differences in PD-L1 mRNA expression occurred with respect to gender, age, KPS score, differentiation, number of metastatic organs, liver metastasis, and peritoneal metastasis (*P* > 0.05). Positive PD-L1 mRNA expression in patients with gastroesophageal junction exceeded that of patients with non-gastroesophageal junction, but this was not statistically significant (*P* = 0.054; Table 2). Besides, we found that PD-L1 expression was higher in intestinal type than in diffuse type (*P* = 0.010; Table 2).

**Table 2:** Correlation of PD-L1 mRNA expression to clinicopathological characteristics

Characteristics	PD-L1 mRNA expression		*P*
	Positive (%)	Negative (%)	
Gender			
Male	46 (34.3%)	88 (65.7%)	0.826
Female	10 (32.3%)	21 (67.7%)	
Age (years)			
≥ 65	18 (43.9%)	23 (56.1%)	0.120
< 65	38 (30.6%)	86 (69.4%)	
KPS			
60–70	5 (50.0%)	5 (50.0%)	0.268
80–100	51 (32.9%)	104 (67.1%)	
Differentiation^*^			
Well	14 (37.8%)	23 (62.2%)	0.570
Poor	42 (32.8%)	86 (67.2%)	
Lauren classification			
Intestinal	13 (56.5%)	10 (43.5%)	0.010
Diffuse	13 (25.5%)	38 (74.5%)	
Primary site			
Non-gastroesophageal junction	30 (28.6%)	75 (71.4%)	0.054
Gastroesophageal junction	26 (43.3%)	34 (56.7%)	
Number of metastatic organs			
≥ 3	18 (32.1%)	38 (67.9%)	0.727
< 3	38 (34.9%)	71 (65.1%)	
Liver metastasis			
Yes	27 (34.2%)	52 (65.8%)	0.951
No	29 (33.7%)	57 (66.3%)	
Peritoneal metastasis			
Yes	8 (33.3%)	16 (66.7%)	0.946
No	48 (34.0%)	93 (66.0%)	

Note:

* well, including high-differentiation and middle-differentiation adenocarcinoma; poor, including low-differentiation adenocarcinoma, mucinous adenocarcinoma, and signet-ring cell carcinoma.

### Association of PD-L1 mRNA expression with clinical response and survival

Among all subjects, 93.3% had clinical response evaluations. Patients with partial response (PR), stable disease (SD), and progressive disease (PD) who positively expressed PD-L1 mRNA were 33.9%, 53.6%, and 7.1%, respectively, and these were not significantly different from those (PR, 40.4%; SD, 36.7%; PD, 15.6%) who were negative for PD-L1 mRNA expression. Objective response and disease control rates of both groups were 33.9%, 87.5% and 40.4%, 77.1%, respectively (*P* > 0.05).

**Figure 3:**
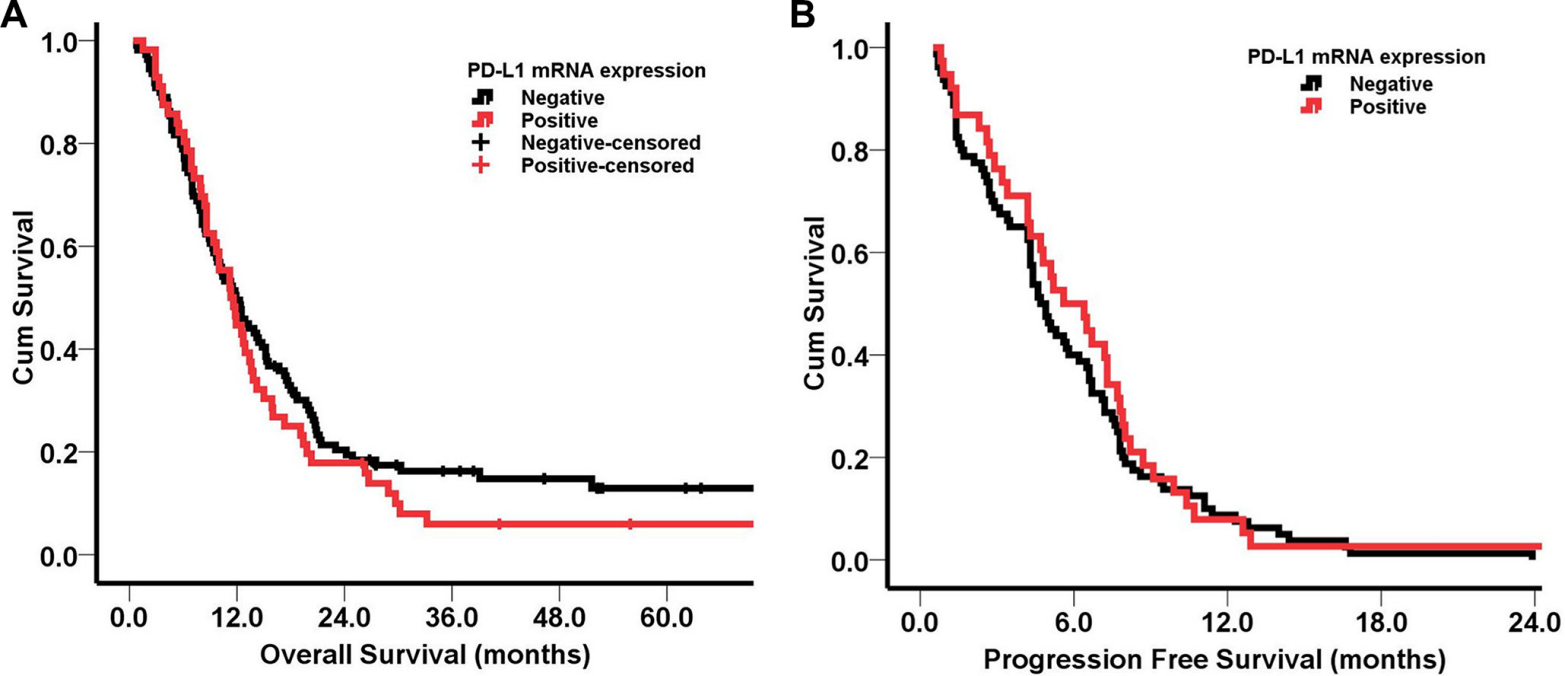
Kaplan-Meier survival curves according to PD-L1 mRNA expression (**A**) OS of patients with PD-L1 mRNA positive and negative expressions; (**B**) PFS of patients with PD-L1 mRNA positive and negative expressions. No significant differences of OS and PFS were found between patients with PD-L1 mRNA positive and negative expressions.

The median OS of patients with PD-L1 mRNA positive and negative expression was 11.3 months (95% CI = 9.0–13.6) and 11.9 months (95% CI = 9.5–14.3), respectively (*P* > 0.05; Figure 3A). Moreover, the median PFS of first-line chemotherapy for patients with PD-L1 mRNA positive and negative expression was 5.6 months (95% CI = 3.5–5.7) and 4.7 months (95% CI = 4.0–5.4), respectively (*P* > 0.05; Figure 3B).

## DISCUSSION

Immunotherapy is promising for cancer [16], and has offered PD-1 and PD-L1 therapeutic targets [17]. At present, anti-PD-1 therapy (nivolumab) is a successful squamous-cell NSCLC and metastatic melanoma treatment [18], however, the significance of tumor-mediated PD-L1 expression is controversial. Brahmer's group reported that expression of PD-L1 was neither prognostic nor predictive of benefit in squamous-cell NSCLC [6]; however, Larkin's group suggested that metastatic melanoma patients with positive PD-L1 expression may benefit from anti-PD-1 therapy [19]. Many studies confirmed PD-L1 expression with IHC of FFPE sections, and no uniform standard was defined for PD-L1 positivity. Brahmer and coworkers defined PD-L1 positivity by staining of the tumor-cell membrane (at any intensity) in 1%, 5%, or 10% of cells, however, Larkin reported that PD-L1 positivity required at least 5% of tumor cells to stain for PD-L1 with any intensity [19]. Therefore, accurate determination of PD-L1 expression by IHC is limited due to few reliable antibodies, interpretative objectivity, and positivity thresholds.

Here, we detected PD-L1 expression in advanced gastric cancer using IHC in protein level and RNAscope assay in mRNA level, which was an antibody-independent assay for *in situ* PD-L1 mRNA detection of FFPE sections, using a rationale similar to that used to confirm HER2 amplification [15]. PD-L1 mRNA signals in AGC were located either in tumor cells or in the mesenchyme, and they were heterogeneous brown dotted or clustered patterns. Using this reproducible and quantitative method, PD-L1 positive mRNA expression was defined as PD-L1 expression in the mesenchyme or ≥ 1% tumor cells according to KEYNOTE-012 trial data [14]. PD-L1-positive expression was found in 33.9% and 35.1% patients in mRNA level and protein level, respectively.

Alba's group compared RNA *in situ* hybridization (RNA-ISH), fluorescent *in situ* hybridization (FISH), and IHC for HER2 amplification and expression in breast cancer [20]. Data show that the concordance between HER2 mRNA expression by RNA-ISH and HER2 amplification by FISH or HER2 protein expression by IHC was 96.5% and 95.2%, respectively. In breast cancer and NSCLC, PD-L1 mRNA was positively non-linear in relationship with PD-L1 protein, suggesting a potential application of RNAscope assay for future clinical studies [12, 13]. In our study, a positive relationship was also found between PD-L1 mRNA and PD-L1 protein. Compared to IHC, RNAscope assay could provide an intuitional and quantitative data. These evidences suggested that RNA *in situ* hybridization using RNAscope assay may hold promise for future research due to its specificity, reproducible, and interpretative objectivity.

PD-L1 expression has been associated with clinicopathological characteristics and prognosis in different cancers [21–24]. We found no significant difference between PD-L1 mRNA expression and gender, age, KPS score, differentiation, the number of metastatic organs, liver metastasis, and peritoneal metastasis (*P* > 0.05). PD-L1 mRNA expression in patients with gastroesophageal junction exceeded that of patients with non-gastroesophageal junction (43.3% vs. 28.6%), although the difference was not statistically significant (*P* = 0.054). Also, PD-L1 expression was higher in intestinal type than in diffuse type (*P* = 0.010), which suggested the chronic inflammation caused by *H. pylori* infection or EB virus typically related to intestinal type might affect PD-L1 expression. Furthermore, PD-L1 mRNA expression in AGC as not associated with clinical response to first-line chemotherapy, PFS, or OS based on our results.

In conclusion, our findings suggested that the RNAscope assay may be a promising method to detect PD-L1 expression in gastric cancer patients, and might play an important role in clinical trials.

## MATERIALS AND METHODS

### Patients and samples collection

A total of 165 patients with advanced gastric cancer treated in the Department of Gastroenterology of Peking University Cancer Hospital from October 2008 to February 2013 were retrospectively studied. All patients received first-line fluorouracil-based chemotherapy and had FFPE tumor sections containing at least 100 tumor cells prior to chemotherapy. Subjects provided written informed consent for their samples to be used in the research. Clinical data containing clinicopathological characteristics, clinical response and patient survival retrospectively obtained from medical records, and this study was approved by the ethics committees of Peking University Cancer Hospital.

### RNA *in situ* hybridization of PD-L1

PD-L1 mRNAs in FFPE tumor samples were measured with RNAscope assay (Advanced Cell Diagnostics, ACD, Hayward, CA) following the manufacturer's instructions. In brief, 5 μm sections were deparaffinized, incubated with pretreatment reagents 1, 2, and 3 and room temperature for 10 min, boiled for 15 min, 40°C for 30 min, respectively. FFPE samples were hybridized with Hs-CD274-probes (ACD) at 40°C for 2 h. Hybridization signals were amplified and visualized with RNAscope 2.0 HD detection kit (Brown). Images were captured with a fluorescent microscope (Nikon ECLIPSE 80i) and the data were analyzed either by scoring or using Spot Studio software.

**Figure 4:**
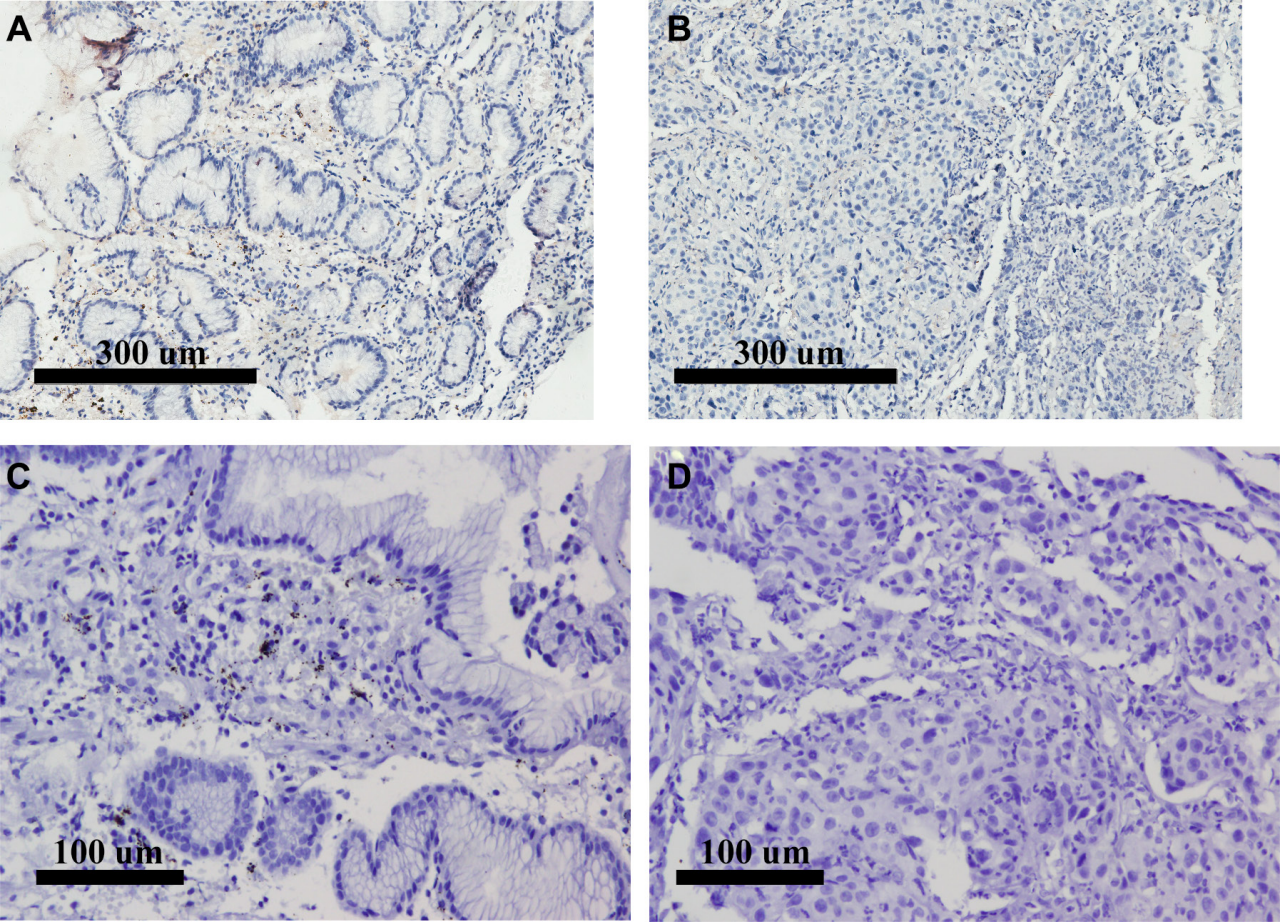
FFPE RNAscope assays using RNAscope^®^2.0 HD Detection Kit (Brown) compared to IHC of the same samples (**A**) Positive IHC of PD-L1 under 100× magnification; (**B**) Negative IHC of PD-L1 under 100× magnification; (**C**) Positive Probe of Hs-PPIB hybridization of the same sample in A under 200× magnification; (**D**) Negative Probe of DapB hybridization of the same sample in B under 200× magnification.

RNAscope results were examined under a standard bright field microscope at 200–400× magnification. Positive signals showed as brown punctuate dots. PPIB and DapB were positive and negative probes, respectively, to control tissue RNA conditions and non-specific hybridization. Figure 4 shows that DapB and Hs-PPIB hybridization of FFPE sections were captured at 200× magnification, which compared to PD-L1 IHC of the same sample. After image capture, RNAscope positive signals were quantified using RNAscope® Spot Studio Software (ACD) which provides statistics for cell-count/region and number of spots/cell (Supplementary Figure S1).

### Immunohistochemistry staining of PD-L1

PD-L1 protein expression was determined by immunohistochemistry (IHC) assay using anti-PD-L1 antibody (ab58810, 1:100, Abcam) according to previous procedure [25]. Briefly, after deparaffinating, hydrating, retrieval, and endogenous peroxidase treatment, FFPE sections were incubated with PD-L1 antibody for 60 min followed by signal production. Sections were scored by two pathologists in the department of pathology at Peking University Cancer Hospital who were blinded to this study. Staining was graded as 0, 1+, 2+, and 3+ if < 1%, ≥ 1% but < 5%, ≥ 5% but < 10%, or ≥ 10% of cells per area were PD-L1 positive, respectively, according to the published criteria [26]. The expression level of PD-L1 was considered positive or negative based on the median staining score.

### Statistical analysis

SPSS 16.0 software was used for statistical analysis. The association between PD-L1 expressions in mRNA level and protein level was analyzed by Kappa and McNemar's tests. The associations between PD-L1 expression and clinical characteristics were analyzed using a Chi-square test or Fisher's exact test. Kaplan-Meier survival curves and a log-rank test were used to assess patient survival. *P* < 0.05 with two-sided test was considered statistical significant.

## SUPPLEMENTARY MATERIALS AND FIGURES



## References

[1] Price TJ, Shaprio JD, Segelov E, Karapetis CS, Pavlakis N, Van Cutsem E, Shah MA, Kang YK, Tebbutt NC (2012). Management of advanced gastric cancer. Expert Rev Gastroenterol Hepatol.

[2] Pardoll DM (2012). The blockade of immune checkpoints in cancer immunotherapy. Nat Rev Cancer.

[3] Freeman GJ, Long AJ, Iwai Y, Bourque K, Chernova T, Nishimura H, Fitz LJ, Malenkovich N, Okazaki T, Byrne MC, Horton HF, Fouser L, Carter L (2000). Engagement of the PD-1 immunoinhibitory receptor by a novel B7 family member leads to negative regulation of lymphocyte activation. J Exp Med.

[4] Sznol M, Chen L (2013). Antagonist antibodies to PD-1 and B7-H1 (PD-L1) in the treatment of advanced human cancer. Clin Cancer Res.

[5] Chen L, Flies DB (2013). Molecular mechanisms of T cell co-simulation and co-inhibition. Nat Rev Immunol.

[6] Brahmer JR, Tykodi SS, Chow LQ, Hwu WJ, Topalian SL, Hwu P, Drake CG, Camacho LH, Kauh J, Odunsi K, Pitot HC, Hamid O, Bhatia S (2012). Safety and activity of anti-PD-L1 antibody in patients with advanced cancer. New Engl J Med.

[7] Reiss KA, Forde PM, Brahmer JR (2014). Harnessing the power of the immune system via blockade of PD-1 and PD-L1: a promising new anticancer strategy. Immunotherapy.

[8] Hino R, Kabashima K, Kato Y, Yagi H, Nakamura M, Honjo T, Okazaki T, Tokura Y (2010). Tumor cell expression of programmed cell death-1 ligand 1 is a prognostic factor for malignant melanoma. Cancer.

[9] Philips GK, Atkins M (2015). Therapeutic uses of anti-PD-1 and anti-PD-L1 antibodies. International Immunology.

[10] Merelli B, Massi D, Cattaneo L, Mandata M (2014). Targeting the PD-1/PD-L1 axis in melanoma: biological rationale, clinical challenges and opportunities. Crit Rev Oncol Hematol.

[11] Gadiot J, Hooijkaas AI, Kaiser AD, van Tinteren H, van Boven H, Blank C (2010). Overall survival and PD-L1 expression in metastasized malignant melanoma. Cancer.

[12] Schalper KA, Velcheti V, Carvajal D, Wimberly H, Brown J, Pusztai L, Rimm DL (2014). *In situ* tumor PD-L1 mRNA expression is associated with increased TILs and better outcome in breast carcinoma. Clin Cancer Res.

[13] Velcheti V, Schalper KA, Carvajal DE, Anagnostou VK, Syrigos KN, Sznol M, Herbst RS, Gettinger SN, Chen L, Rimm DL (2014). Programmed death ligand-1 expression in non-small cell lung cancer. Lab Invest.

[14] Muro K, Bang Y-J, Shankaran V, Geva R, Virgil D, Catenacci T, Gupta S, Eder JP, Berger R, Gonzalez EJ, Ray A, Dolled-Filhart M, Emancipator K (2015). Relationship between PD-L1 expression and clinical outcomes in patients(Pts) with advanced gastric cancer treated with the anti-PD-1 monoclonal antibody pembrolizumab (Pembro: MK-3475) in KEYNOTE-012. J Clin Oncol.

[15] Hofmann M, Stoss O, Shi D, Büttner R, van de Vijver M, Kim W, Ochiai A, Rüschoff J, Henkel T (2008). Assessment of a HER2 scoring system for gastric cancer: results from a validation study. Histopathology.

[16] Sharon E, Streicher H, Goncalves P, Chen HX (2014). Immune checkpoint inhibitors in clinical trials. Chin J Cancer.

[17] Sznol M (2014). Blockade of the B7-H1/PD-1 pathway as a basis for combination anticancer therapy. Cancer J.

[18] Topalian SL, Hodi FS, Brahmer JR, Gettinger SN, Smith DC, McDermott DF, Powderly JD, Carvajal RD, Sosman JA, Atkins MB, Leming PD, Spigel DR, Antonia SJ (2012). Safety, activity, and immune correlates of anti-PD-1 antibody in cancer. N Engl J Med.

[19] Larkin J, Chiarion-Sileni V, Gonzalez R, Grob JJ, Cowey CL, Lao CD, Schadendorf D, Dummer R, Smylie M, Rutkowski P, Ferrucci PF, Hill A, Wagstaff J (2015). Combined Nivolumab and Ipilimumab or monotherapy in Untreated melanoma. N Engl J Med.

[20] Alba J, Gutierrez J, Coupe VM, Fernández B, Vázquez-Boquete Á, Alba J, Forteza J, García-Caballero T (2012). HER2 status determination using RNA-ISH—a rapid and simple technique showing high correlation with FISH and IHC in 141 cases of breast cancer. Histol Histopathol.

[21] Qing Y, Li Q, Ren T, Xia W, Peng Y, Liu GL, Luo H, Yang YX, Dai XY, Zhou SF, Wang D (2015). Upregulation of PD-L1 and APEI is associated with tumorigenesis and poor prognosis of gastric cancer. Drug Des Devel Ther.

[22] Gao Q, Wang XY, Qiu SJ, Yamato I, Sho M, Nakajima Y, Zhou J, Li BZ, Shi YH, Xiao YS, Xu Y, Fan J (2009). Overexpression of PD-L1 significantly associates with tumor aggressiveness and postoperative recurrence in human hepatocellular carcinoma. Clin Cancer Res.

[23] Song M, Chen D, Lu B, Wang C, Zhang J, Huang L, Wang X, Timmons CL, Hu J, Liu B, Wu X, Wang L, Wang J (2013). PTEN loss increases PD-L1 protein expression and affects the correlation between PD-L1 expression and clinical parameters in colorectal cancer. PLoS One.

[24] Wu C, Zhu Y, Jiang J, Zhao J, Zhang XG, Xu N (2006). Immunohistochemical localization of programmed death-1 ligand-1 (PD-L1) in gastric carcinoma and its clinical significant. Acta Histochem.

[25] Yu J, Gao J, Lu Z, Gong J, Li Y, Dong B, Li Z, Zhang X, Shen L (2014). Combination of microtubule associated protein-tau and β-tubulin III predicts chemosensitivity of paclitaxel in patients with advanced gastric cancer. Eur J Cancer.

[26] Herbst RS, Soria JC, Kowanetz M, Fine GD, Hamid O, Gordon MS (2014). Predictive correlates of response to the anti-PD-L1 antibody MPDL3280A in cancer patients. Nature.

