# Supplementary material for: Programmed death-ligand-1 expression in advanced gastric cancer detected with RNA in situ hybridization and its clinical significance
Source: Oncotarget. 2016 May 15;7(26):39671–9. doi: 10.18632/oncotarget.9381 (PMC5129961; doi:10.18632/oncotarget.9381)
Supplement: Supplementary file 1 [file oncotarget-07-39671-s001.pdf]

Programmed death-ligand-1 expression in advanced gastric cancer detected with RNA *in situ* hybridization and its clinical significance

Supplementary Materials

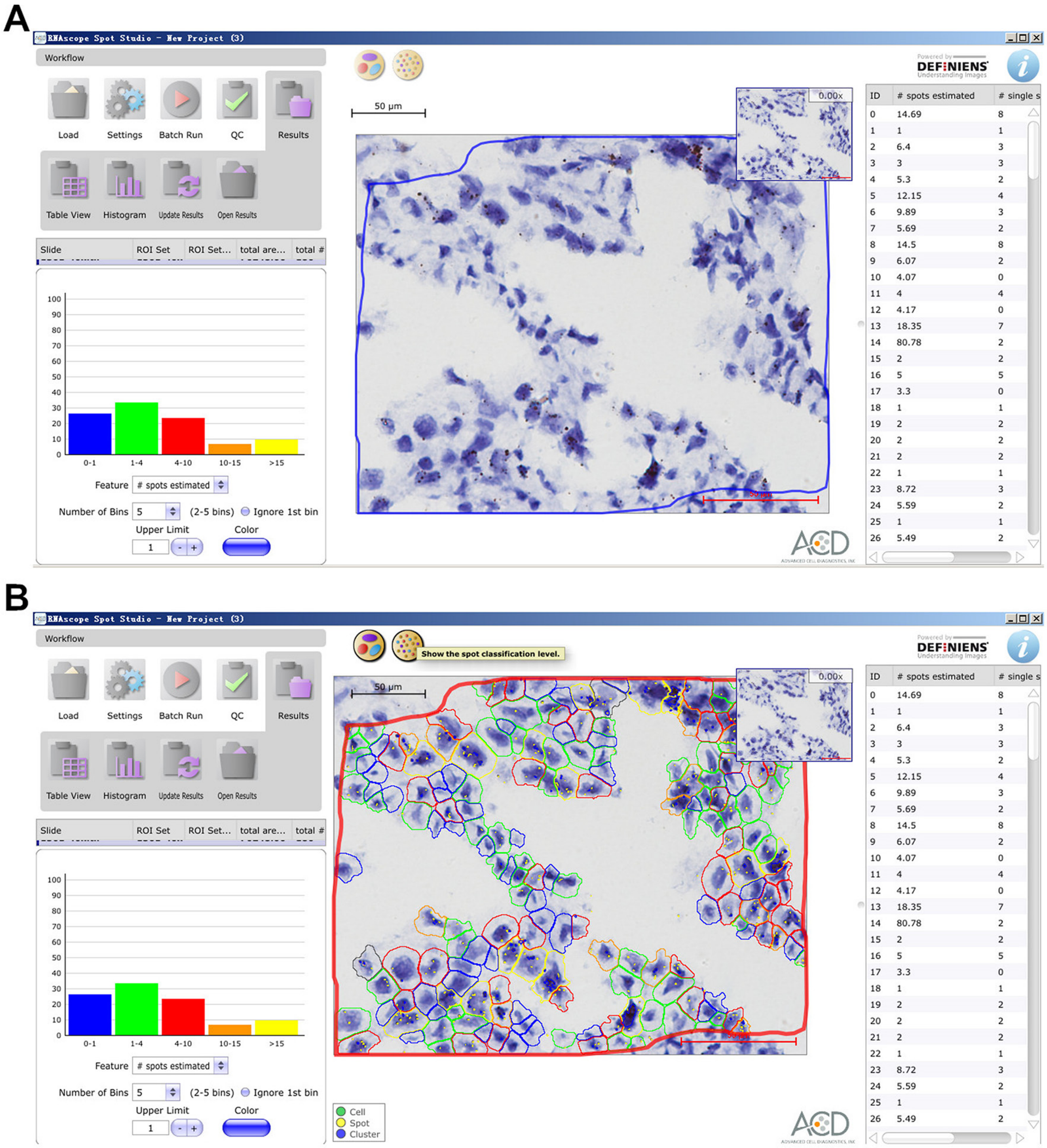

Supplementary Figure S1: The analyzed result using RNAscope® Spot Studio Software. (**A**) The original captured image under 400× magnification was input into the software according to the protocol of software; (**B**) After image was input into the software, cells with different brown dots were circled using different colors followed by cell counting with different colors as shown in histogram. Cell numbers with 0–1 dot / cell, 1–4 dots / cell, 4–10 dots / cell, 10–15 dots / cell, and > 15 dots / cell were presented in blue, green, red, orange, and yellow bar, respectively.

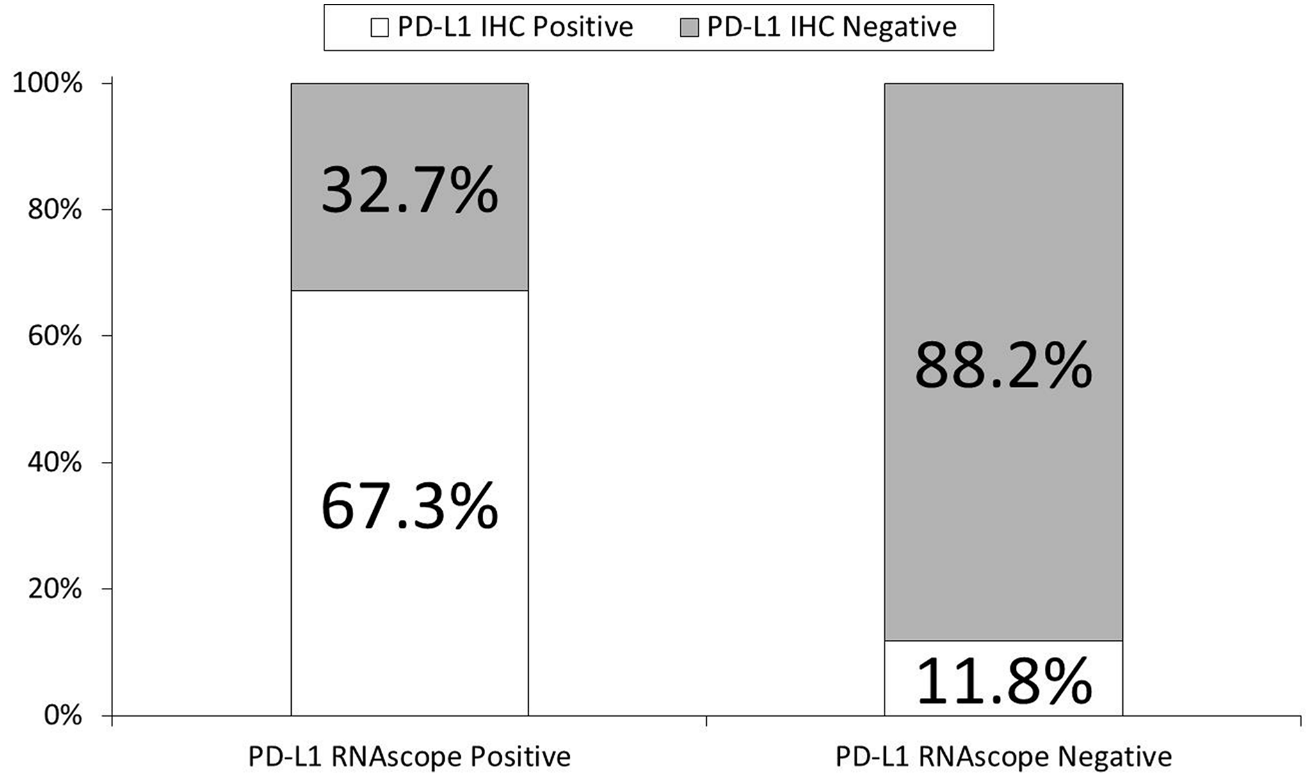

Supplementary Figure S2: Relationship between PD-L1 protein expression and PD-L1 mRNA expression.
